# A Deep Neural Network for Accurate and Robust Prediction of the Glass Transition Temperature of Polyhydroxyalkanoate Homo- and Copolymers

**DOI:** 10.3390/ma13245701

**Published:** 2020-12-14

**Authors:** Zhuoying Jiang, Jiajie Hu, Babetta L. Marrone, Ghanshyam Pilania, Xiong (Bill) Yu

**Affiliations:** 1 Department of Civil and Environmental Engineering, Case Western Reserve University, Cleveland, OH 44106, USA; zxj45@case.edu; 2 Departments of Computer and Data Sciences, and Electrical, Computer, and Systems Engineering, Case Western Reserve University, 2104 Adelbert Road, Cleveland, OH 44106, USA; jxj919@case.edu; 3 Bioscience Division, Los Alamos National Laboratory, Los Alamos, NM 87545, USA; blm@lanl.gov; 4 Materials Science and Technology Division, Los Alamos National Laboratory, Los Alamos, NM 87545, USA; gpilania@lanl.gov

**Keywords:** molecular fingerprint, deep neural network, glass transition temperature, copolymers, quantitative structure–property relationship (QSPR)

## Abstract

The purpose of this study was to develop a data-driven machine learning model to predict the performance properties of polyhydroxyalkanoates (PHAs), a group of biosourced polyesters featuring excellent performance, to guide future design and synthesis experiments. A deep neural network (DNN) machine learning model was built for predicting the glass transition temperature, *T*_g_, of PHA homo- and copolymers. Molecular fingerprints were used to capture the structural and atomic information of PHA monomers. The other input variables included the molecular weight, the polydispersity index, and the percentage of each monomer in the homo- and copolymers. The results indicate that the DNN model achieves high accuracy in estimation of the glass transition temperature of PHAs. In addition, the symmetry of the DNN model is ensured by incorporating symmetry data in the training process. The DNN model achieved better performance than the support vector machine (SVD), a nonlinear ML model and least absolute shrinkage and selection operator (LASSO), a sparse linear regression model. The relative importance of factors affecting the DNN model prediction were analyzed. Sensitivity of the DNN model, including strategies to deal with missing data, were also investigated. Compared with commonly used machine learning models incorporating quantitative structure–property (QSPR) relationships, it does not require an explicit descriptor selection step but shows a comparable performance. The machine learning model framework can be readily extended to predict other properties.

## 1. Introduction

With the rapid development of civilization, the demand for plastic products has increased significantly. However, plastic waste has become a worldwide environmental issue because plastic materials are durable and difficult to degrade [1,2,3]. As a result, bioplastics, which are made from biological substances, have attracted significant interest as an alternative for conventional plastics made from petroleum [4,5]. Polyhydroxyalkanoate (PHA) polymers are a type of green bioplastics that are produced naturally by bacteria and utilized by the cells for energy and carbon storage [6]. PHAs have been used commercially for a wide range of applications due to their excellent versatility, biocompatibility, and biodegradability Their application areas range from food packaging and agricultural films to biomedical fields including use as drug carriers, tissue engineering scaffolds, etc. [7,8,9]. 

To fulfill the requirements for various applications, fabrication of PHAs with a variety of properties, such as physical, thermal, and mechanical properties, becomes a new challenge. PHAs with different properties are achieved by tuning the monomers and configurations [10,11,12]. It is known that approximately 150 types of PHA monomers can be used to constitute different PHA copolymers [13]. These combinations of single monomers can provide PHA polymers with diverse and flexible properties. In addition, the polymer properties can also be tuned by modifying chemical configurations, i.e., the size, shape, and branching of polymers [14,15]. Therefore, the design and synthesis of novel PHAs typically need trial and error experimentation to obtain the structure–property–performance relationships. This traditional approach requires enormous lab and labor investments, and progress is typically slow.

As data science has rapidly developed in the last decade, machine learning (ML) became a promising tool for data analytics and predictions. ML is part of data science and it is a technology for processing a large amount of data. It learns from the existing data, finds data patterns, and provides solutions [16]. It is faster, cheaper, and more flexible than experimentation. A neural network is an algorithm of ML. Inspired by the neural network of the human brain, an artificial neural network can learn from past data and generate a response [17]. The structure of a typical artificial neural network is usually composed of three essential types of layers, i.e., the input layer, hidden layer(s), and output layer. They are used for receiving, processing, and exporting information, respectively. Each neuron is connected with an assigned weight and the weight sum is calculated by a transfer function [18]. Deep learning is a class of ML that allows multiple hidden layers for data processing [19]. A deep neural network (DNN) combines an artificial neural network with deep learning and is capable of providing a better solution to problems in cognitive learning such as speech and image recognition [20]. So far, DNN models have been successfully applied to learn and predict a range of properties of diverse types of materials, including metals, ceramics, and macromolecular materials [21,22,23].

Due to the increasing demand for plastic materials and rising awareness of the plastic waste crisis, PHAs have been intensively explored since their first discovery in 1926 [24]. With tremendous historical experimental efforts focused on PHA synthesis and characterization, a large amount of data is accessible from published sources. Therefore, this allows data to be extracted from the literature and used to build a data-based ML model to investigate the connections between structures and properties of PHAs and to extract design rules and useful chemical trends.

This study is a follow-up to [25], in which an ML model incorporating descriptors extracted from the quantitative structure–property relationship (QSPR) for glass transition temperature (*T*_g_) prediction was used. It describes the development of a DNN-based model for *T*_g_ predictions of PHA homo- and copolymers. We used *T*_g_ as the predicted property of this model because *T*_g_ is an important thermal property for polymers turning from a rigid state to a rubbery state. *T*_g_ has been extensively studied and its value depends on the structure of polymers, e.g., molecular weight and branching network [26,27]. The database consists of 133 data points obtained from published experiments [25]. The DNN-based model was used to investigate the connections between the inputs, i.e., structure of PHA polymers, and the output, i.e., *T*_g_. The trained model can provide guidance for material design of PHA polymers before conducting experiments, which can significantly reduce experimental trials, and, therefore, save time and expense. 

The novelty of this work is the prediction of the glass transition temperature using a DNN model that is simple to implement and still achieves accurate results. Compared with prior ML model approach (i.e., [25]), this model does not require the assistance of an expert to obtain the chemical descriptors from QSPR; instead, it uses standard molecular fingerprints to convert the polymers into machine-readable language. This is not only much easy to encode the copolymers but also avoids possible cognitive bias in QSPR by different professionals. This ML model framework can also be easily extended to a broader range of polymers beyond PHA copolymers. 

## 2. Materials and Methods

### 2.1. Data Preparation

**Figure 1:**
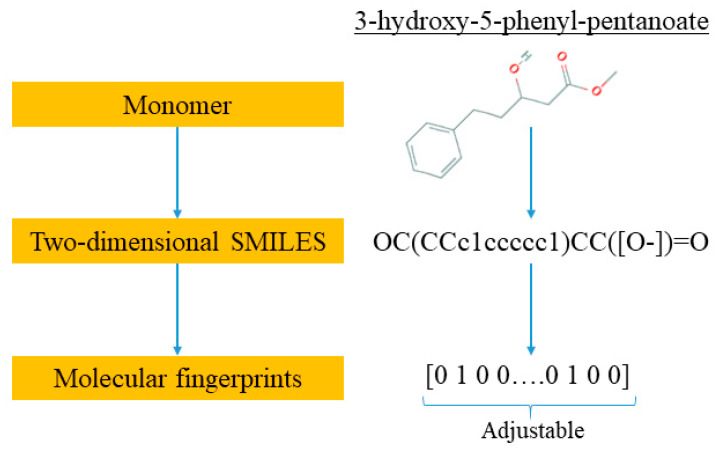
An example illustration of steps in encoding a chemical with digital representation to generate a molecular fingerprint.

The experimental measurements of *T*_g_ of a variety of polyhydroxyalkanoate-based homo- and copolymers were obtained from the work of Pilania et al. by one of the coauthors [25]. The experimental database of the PHA *T*_g_ values is cooling-rate-dependent and based on the metastable/dynamic behavior of amorphous polymer systems. The experimental measurements were made using the differential scanning calorimetry (DSC) technique with relatively slow cooling rates (~10–30 K/min). The *T*_g_ data set is provided in the Supplementary Materials in Table S1. The database included 133 data points and each data source contained five variables, i.e., chemical identities of monomer A and monomer B, the fractional composition of monomer A, the polymer molecular weight (MW) with a unit of kg mol^−1^, and polydispersity index (PDI). They were set as the inputs of the DNN model and the output was *T*_g_. To convert the chemical expressions of monomer A and B into machine-readable information, the monomer names were first transformed into two-dimensional SMILES strings [28]. SMILES represents the simplified molecular input line entry system; it describes the unique structure and chemical species of a chemical compound. The chemical identifier resolver (https://cactus.nci.nih.gov) was used for the conversion. The second step was to transform the SMILES strings into machine-readable data. Molecular fingerprinting is an approach to encode a chemical into a binary digit vector, e.g., {0 0 1 … 1 1 0}, which represents molecules in digital representation readable by a computer. The length of the vector is adjustable, which could significantly affect the reliability of the DNN model. Therefore, a model optimization was conducted to select the best value of the vector length as discussed in detail in the next subsection. In this work, the RDKit package (http://www.rdkit.org) was used to convert SMILES into molecular fingerprints [29]. An example of a monomer, i.e., 3-hydroxy-5-phenyl-pentanoate, is illustrated for this two-step conversion in Figure 1. For a homopolymer, monomer B was set to an all zero array and the fractional composition of A in the polymer was 100%. All the monomers were subjected to the encoding process as illustrated in Figure 1 to produce the corresponding digital representation via molecular fingerprints.

### 2.2. DNN Model Construction

**Figure 2:**
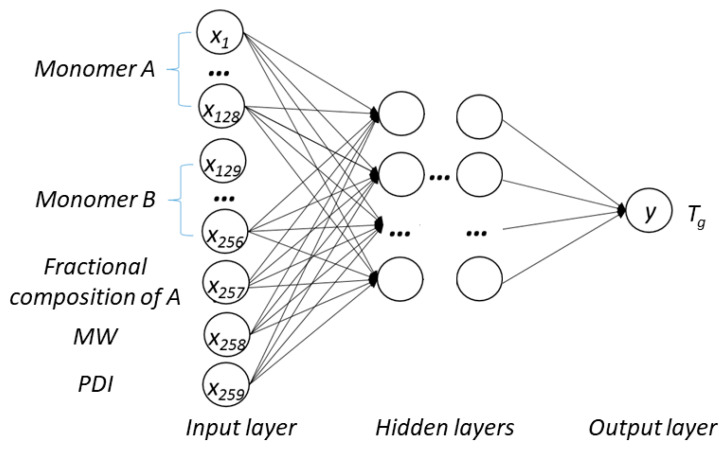
Illustration of the deep neural network (DNN) model structure.

A DNN model was built to learn and predict the relationship between the input variables and an output. Five input variables included the molecular fingerprints of monomer A and B, the fractional composition of monomer A, molecular weight, and PDI. The output was *T*_g_. Figure 2 shows the schematic structure of the DNN model. The monomers A and B were converted to molecular fingerprints before being entered into the model. The optimal length of the molecular fingerprints was determined using a Bayesian optimization scheme. After conversion to the molecular fingerprints, monomers A and B are each represented by a vector that is fed into the corresponding number of neurons, i.e., as the number of neurons to accept the molecular fingerprint inputs is equal to the length of the molecular fingerprint employed. Each of the other three numerical inputs, i.e., fractional composition of monomer A, molecular weight, and PDI, occupied one neuron. Therefore, the total number of neurons in the input layer equals the 2*N+3, where N is the length of the molecular fingerprints. Rectified linear unit (ReLU) was used as a nonlinear transfer function and the loss function used was the mean square error (MSE) of the measured glass transition temperature *T*_g_. The learning rate was chosen to be a cosine annealing learning rate, which reduces the learning rate according to a half cosine curve. The optimization function used was Adam [30]. The dropout technique was also used to avoid overfitting of the training data.

To obtain a reliable DNN model structure, the hyperparameters of the model (i.e., the number of neurons and the number of layers) were optimized with a Bayesian optimization scheme. Bayesian optimization works by constructing a posterior distribution of functions, i.e., a Gaussian process, that best describes the target function. With the increase in the number of hyperparameter combinations observed, the posterior distribution is improved, and the algorithm becomes more certain of which hyperparameter combination should be picked for the next observation. This process is designed to minimize the number of steps required to find a combination of hyperparameters that are close to the optimal combination [31]. From these, the length of the molecular fingerprints of the two monomers and the structure of the hidden layer, i.e., the number of layers and neurons in each layer, and dropout rate, i.e., the percentage of neurons to be ignored during training, were determined. To start the hyperparameter optimization process, the ranges of the hyperparameters were first defined. It was then subjected to the Bayesian optimization process that automatically identifies the optimal set of hyperparameters. The hyperparameters of the DNN are optimized after this process. The resultant optimal hyperparameters lead to the length of the molecular fingerprints as 128, the number of hidden layers set as 7, the number of neurons in each hidden layer set as 512, and the dropout rate set as 0.2.

To augment the limited data set, the DNN model was trained and tested via a fivefold cross-validation method. This method first randomly partitioned the data into five nearly equal-sized subgroups. Of the five subgroups, a single subgroup was retained for testing, and the remaining four subgroups were used for training. This process was then repeated five times, with each of the subgroups used exactly once for model testing. Such a process effectively augmented the data by five times. The prediction results of each testing subgroup were obtained, and the overall accuracy was calculated by averaging the sum of the evaluating scores. The training epoch was set as 500 to ensure convergence.

## 3. Results and Discussion

### 3.1. Performance of the DNN Model

**Figure 3:**
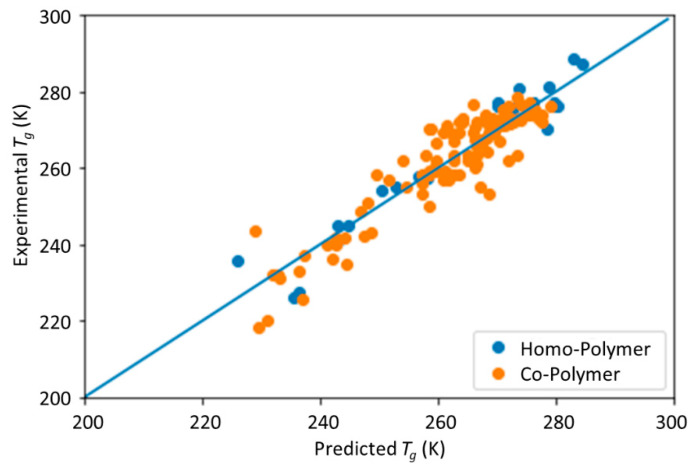
The scatter plot of the predicted glass transition temperature, *T*_g_ by the DNN model vs. experimental measurement results, *T*_g_ on the test data.

The scatter plot of the predicted vs. experimental *T*_g_ data is shown in Figure 3. The homopolymers are labeled in orange and the copolymers are labeled in blue. The accuracy and reliability of the DNN model was evaluated by the coefficient of determination (*R*^2^), mean absolute error (MAE), and root mean square error (RMSE). The *R*^2^, MAE, and RMSE of each subgroup and their averaged scores using the fivefold cross-validation method are listed in Table 1. From Table 1, it is seen that the *R*^2^, MAE, and RMSE values are close among each cross-validation subgroup, which indicates that the performance of the DNN model is reliable and robust against small fluctuations in the training data. The overall DNN model had an *R*^2^ of 0.869, MAE of 4.010, and RMSE of 5.339 K. The performance of the DNN model is comparable to the results of the ML model based on QSPR by Pilania et al., which achieved an RMSE of 4.8K using the same data set [25]. A major advantage of this DNN model developed in this study is its simplicity. In the previous study, the initial features of PHAs were first described by a set of QSPR-based features, and then this feature set was further analyzed, compacted, and trained by a random forest ML model. This process requires professional experience and significant domain knowledge in the polymer chemistry. Comparatively, the DNN learning model developed here, which combines the binary fingerprinting with the DNN model, is much more straightforward, easier to implement, and requires minimal expert judgement and therefore is free from preference. In addition, the model can also be easily extended to a broader range of polymers beyond PHA copolymers.

As there is no preference in labeling one of monomers as A and another as B, i.e., the model should contain symmetry, which is not captured in the data set used in the DNN model training shown in Figure 2. For example, regarding monomer A (3H5PhP), monomer B (3H7PhHp) with fractional composition of A at 23% is equivalent to monomer A (3H7PhHp), with monomer B (3H5PhP) having a fractional composition of A at 100% − 23% = 77%. In principle, the machine learning model should capture such symmetry. To evaluate whether the DNN model can capture the symmetry in input data, a new symmetry data set is generated from the original data set by exchanging the monomer A and B and updating the fractional composition of A by 100% fraction composition of A. The molecule weight, PDI, and glass transition temperature remain the same. From physics, the symmetry data set contains the same information of the original data set.

**Figure 4:**
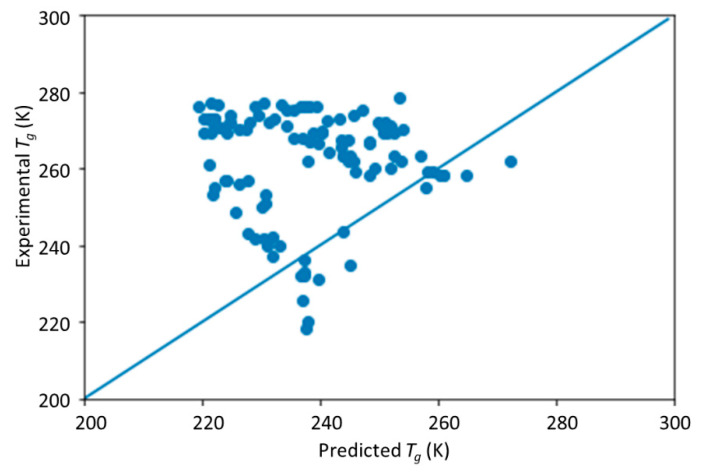
The scatter plot of the predicted vs. experimental *T*_g_ on the symmetry data set by the pretrained DNN model.

The comparison of predicted *T*_g_ by the DNN model versus the measured *T*_g_ on the symmetric data set is shown in Figure 4. Compared with the results shown in Figure 3, the prediction results are much worse. This indicates that the trained DNN model did not capture the symmetry and therefore is sensitive to the order of monomers. This will lead to prediction bias in its application that needs to be improved.

**Figure 5:**
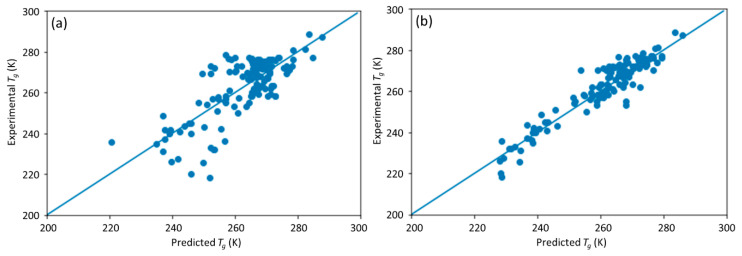
The scatter plot of the predicted vs. experimental *T*_g_ after retraining the DNN model with the combined initial and symmetry data set; (**a**) results with initial data set; (**b**) results with symmetry data set.

To solve this issue, we retrained the model with a combined initial training data set and symmetry data set, using the same five-fold cross-validation method. The overall retrained DNN model had an *R*^2^ of 0.897, MAE of 3.474, and RMSE of 4.748 K. Compared with the initial DNN model, the retrained DNN model achieved higher prediction accuracy. This shows that training the DNN model with a symmetry data set can further improve the model performance. The scatter plots of the predicted *T*_g_ under different data sets are shown in Figure 5. The retrained model achieved similar performance on the initial training data set and the symmetry data set, which proves that incorporating the symmetry data set into the training data set can enable the model to describe the symmetry and be insensitive to the order of monomer A and B designation. Meanwhile, compared with Figure 4, the performance of the retrained DNN model with additional symmetry data set is much better than that trained only with the initial model.

### 3.2. Importance Factors of the Input Variables on T_g_ Prediction 

**Figure 6:**
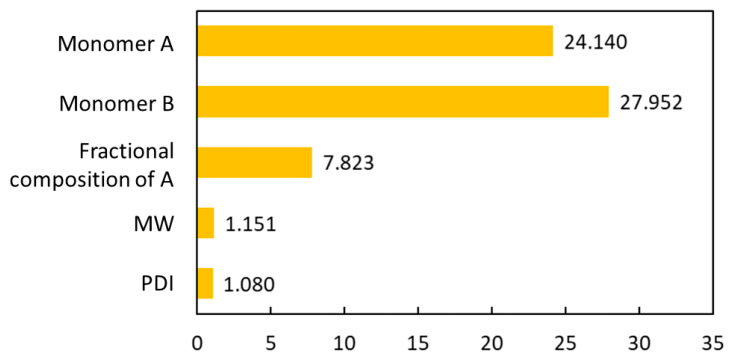
The bar plot of the importance factors of each input variable to the DNN model.

The interpretability of the DNN model was also studied, since understanding why a model makes a certain prediction can be as crucial as the prediction’s accuracy for chemical applications. The DNN model belongs to the category of black-box models. For the interpretability of the DNN model, the SHapley Additive exPlanations (SHAP) method was used to measure the importance of input features to the DNN model. SHAP is a unified framework for interpreting predictions, especially for complex black-box models. It assigns to each feature an importance value for a particular prediction indicating how much a model relies on each of the features—in other words, how much each feature contributes to the prediction. This helps us to understand the impact of input variables on the prediction of *T*_g_ [32]. Figure 6 shows the importance factors of the five variables. It shows that the monomer A and B both had high importance values and that they were of similar importance. This is reasonable since the types of monomer are the most crucial in determining the *T*_g_; in addition, they are interchangeable as input symmetry as discussed earlier. The importance factors of monomer A and B were much higher than the other three variables. This indicates that the chemistry of the two monomers has the predominant effect in determining the *T*_g_ of the PHA polymers. The next one in the importance factor is the fractional composition of A. The relatively low SHAP values of molecular weight and PDI imply that the polymer chain size and dispersity have relatively less influence on *T*_g_ of the PHA polymers as compared to the chemistry of the monomers and the fractional composition of the polymer. 

### 3.3. Comparison with DNN Model with Other ML Methods

**Figure 7:**
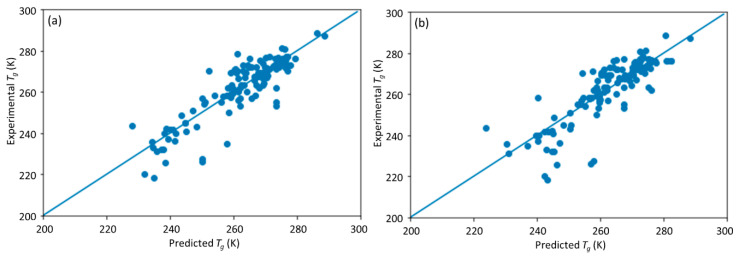
The predicted vs. experimental *T*_g_ using (**a**) SVM and (**b**) LASSO (results by DNN are shown in Figure 5).

The performance of the DNN ML model is compared with other nonlinear ML method such as the support vector machine (SVM) and LASSO, a sparse linear regression model. The results of SVM and LASSO methods on the *T*_g_ prediction have been compared. Fivefold cross-validation was used for all the models. The results are shown in the Table 2 and Figure 7. The Table 2 concludes the *R*^2^, MAE, and RMSE of three methods, i.e., SVM, LASSO, and the DNN model. The DNN model achieved the best performance among these ML models.

### 3.4. DNN Model Prediction Performance with Inaccurate or Missing Information

Although extensive efforts have been devoted to the fabrication of PHA polymers and there are *T*_g_ data on numerous polymers exhibiting a diverse set of chemistries in the literature, some polymer information may be missing or inconsistent for a certain type of PHA. For example, the measurements of molecular weight and PDI are complicated and are prone to error. It is of interest to see how such inaccuracy or missing information would affect the performance of the DNN model prediction and develop proper countermeasures to improve the robustness and accuracy of the DNN model under such circumstances. To evaluate the performance of the developed DNN model to missing values, we assumed one or two inputs were missing for the DNN model, e.g., fractional composition of A only, molecular weight only, PDI only, and molecular weight and PDI both ignored. The performance of different approaches, i.e., directly removing input nodes of the missing values, and replacing the missing data with the mean, maximum, and minimum values, were evaluated on how they affect the model performance.

#### 3.4.1. The Effects of Removing Input Nodes Corresponding to the Missing Values

**Figure 8:**
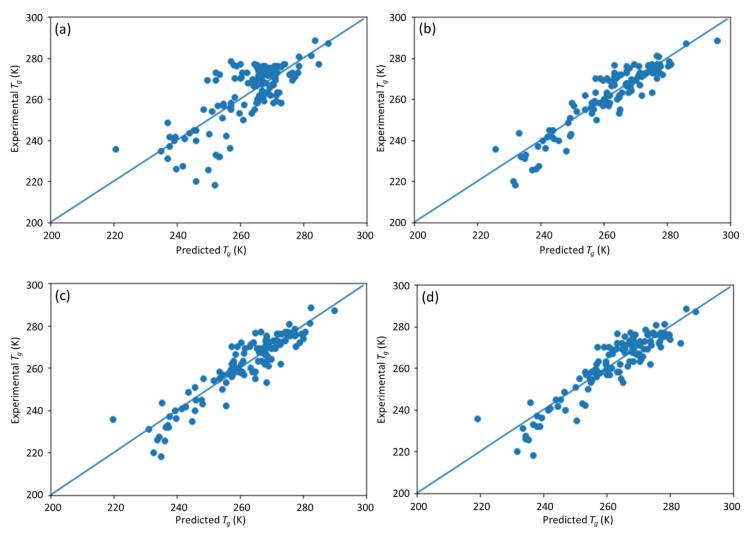
The scatter plot of the predicted vs. experimental *T*_g_ after different inputs were removed from the DNN model. (**a**) Fractional composition of A; (**b**) molecular weight; (**c**) polydispersity index (PDI); (**d**) both molecular weight and PDI.

The direct method to deal with a variable with missing data is to remove the corresponding input nodes to the DNN model. Four sets of DNN models with some input node removed (i.e., without fractional composition of A, without molecular weight, without PDI, and without both the molecular weight and PDI in the feature set) were trained. The prediction results are shown in Figure 8 and the evaluation scores of model accuracy are listed in Table 3. For the case without fractional composition of A, the *R*^2^ of the model dramatically decreased to about 0.5, which indicates that fractional composition of A is a significant factor for *T*_g_ prediction. Indeed, this observation is quite intuitive and can be understood on a physical basis. As the fractional composition determines the chemical composition of the copolymer chemistry, in the absence of this critical feature, all copolymer chemistries formed by combining any two given homopolymers in different compositions essentially become indistinguishable to the model and, as a result, the predictive performance degrades drastically.

Deleting the input node of molecular weight or PDI alone did not significantly compromise the model performance compared with the full-input model. Even when further deleting both molecular weight and PDI, the *R*^2^ was still comparable to the full-input model. These findings indicate that molecular weight and PDI have limited contributions to the final prediction and they are much less important than the chemistry of the monomers and the fractional compositions of monomer A. These are consistent with the results of importance factors of each input. 

#### 3.4.2. The Effects of Replacing the Missing Values with the Mean, Minimum, or Maximum Values

**Figure 9:**
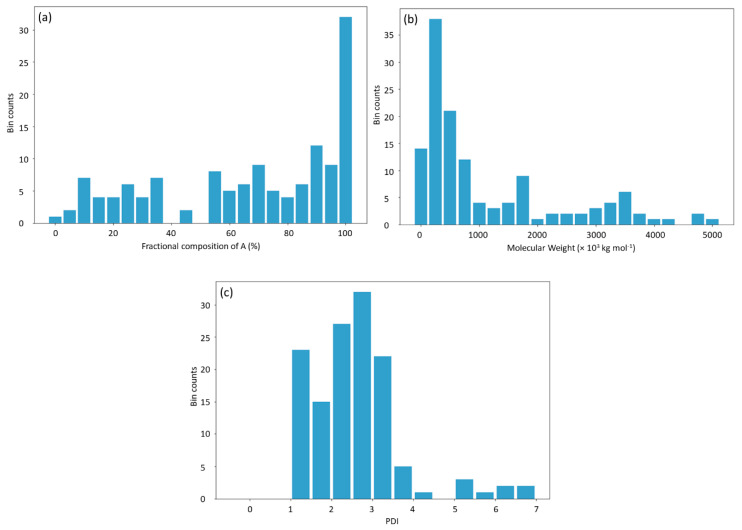
The statistical distribution of the input data. (**a**) Fractional composition of A; (**b**) molecular weight; (**c**) PDI.

An alternative approach to deal with missing data is to replace the missing values with the statistics of training data such as the mean value, the minimum value, or the maximum value. In this way, the models are not required to be retrained; instead, the missing data are replaced with the estimated values obtained from the training data. Figure 9 shows the statistical distribution of the input data. The descriptive statistics of the input data are listed in Table 4.

**Figure 10:**
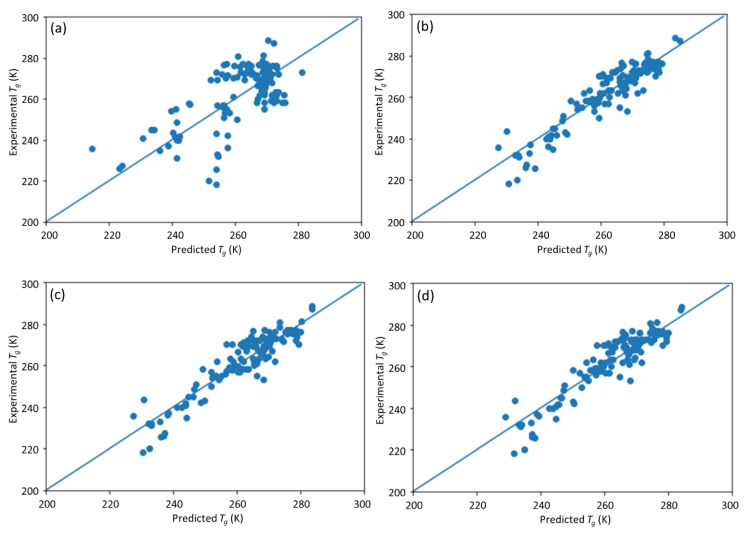
The scatter plot of the DNN model predicted vs. experimental *T*_g_ with different input factors estimated with the mean value. (**a**) Fractional composition of A; (**b**) molecular weight; (**c**) PDI; (**d**) molecular weight and PDI.

A common method to deal with missing data is to replace the missing values with the mean value. Table 5 shows the DNN model prediction performance using this strategy for missing four types of input data, i.e., the fractional composition of A, the molecular weight, the PDI, and both molecular weight and PDI. The scatter plots of the predictions are shown in Figure 10. Compared with the DNN model prediction with full original inputs, replacing the individual inputs with the corresponding mean input for the molecular weight, or PDI, or both, exhibited nearly the same model performance, with the differences of *R*^2^ less than 0.6%. However, an exception is observed for the fractional composition of A, where the DNN model performance with mean value replacing the missing value is worse than simply deleting the input node for the fractional composition A. The observations further validated that the most significant factors for *T*_g_ prediction were the chemistry of the monomers and their fractional compositions in the copolymers. The DNN model still achieved a high degree of accuracy in *T*_g_ prediction without information on the measurement of molecular weight, or PDI, or both of molecular weight and PDI. Such missing information could be estimated with the mean values typical of such data and still achieve decent accuracy.

**Figure 11:**
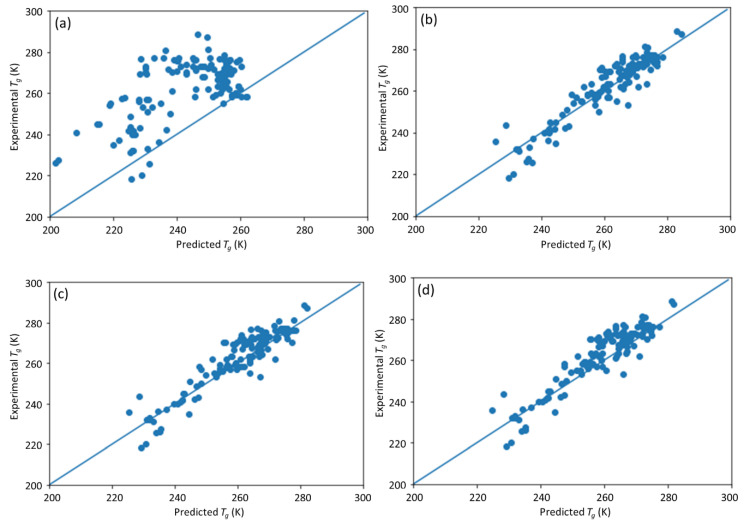
Scatter plots of predicted vs. experimental *T*_g_ with different input factors estimated via their minimal values. (**a**) Fractional composition of A; (**b**) molecular weight; (**c**) PDI; (**d**) molecular weight and PDI.

**Figure 12:**
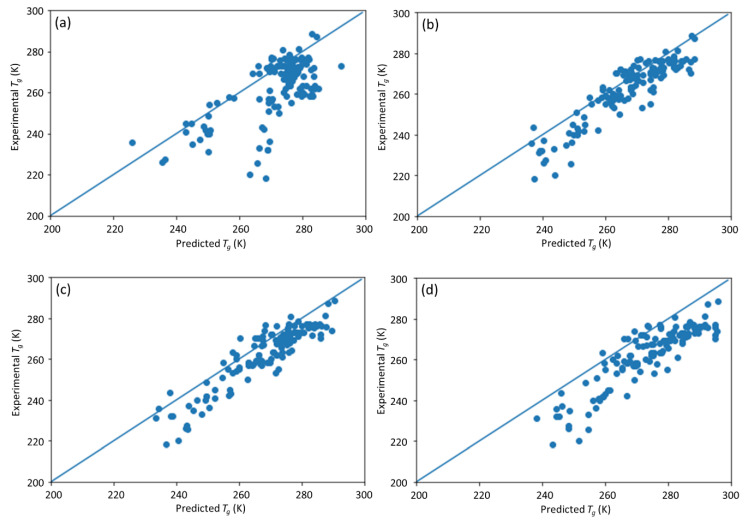
Scatter plots of the predicted vs. experimental *T*_g_ with different input factors estimated with their maximum values. (**a**) Fractional composition of A; (**b**) molecular weight; (**c**) PDI; (**d**) molecular weight and PDI.

To further investigate the sensitivity of the DNN model to the estimation of missing input values, we also analyzed its performance when the missing values are estimated with the minimum or maximum values of the corresponding type of data from the data set. Compared with estimating the missing data with the mean values, estimating the input with the minimum or maximum values can also give a sense of how the model performance is affected by the data quality, since errors might inevitably occur during measurement. Table 6 and Table 7 summarize the model prediction performances when the corresponding input variable is replaced with its minimum or maximum values. Four different conditions were analyzed, i.e., the fractional composition of A, the molecular weight, PDI, and both molecular weight and PDI. The scatter plots of the predictions are shown in Figure 11 and Figure 12. 

Compared with the method of replacing the input variable with the mean value, estimating the input variables with its minimum or maximum values showed a reduced prediction accuracy. In general, the use of minimum values as the estimation led to predictions shifting toward the left of the diagonals or resulted in lower predicted *T*_g_ than measured *T*_g_, while the use of maximum values showed the opposite effect (Figure 12). In terms of the polymer fractional composition of A, as shown in Figure 9a, the minimum value is far away from where the rest of the data are concentrated, compared with the maximum value. This caused the model prediction with the minimal value to be worse than that with the maximum value. In contrast, for MW and PDI, as shown in Figure 9b,c, the maximum value is far away from where the rest of the data are concentrated compared with the minimum value, causing the model prediction with the maximum value to be worse than that with the minimum value. The prediction accuracy by filling missing data with minimal values is only slightly lower than with the mean values for input variables, including the molecular weight, PDI, and molecular weight and PDI, whereas the prediction accuracy by filling missing data with the maximum values drops dramatically. This is possibly due to the fact that the data values follow a skewed statistical distribution (i.e., Figure 9). In such cases, the mean is larger than the median and the maximum value is further away from the mode than the minimum value. Therefore, estimating the missing data with its maximum value leads to a worse prediction than estimating with its minimum value. The results show that the features of missing data are important to proper estimation of missing values to ensure DNN model prediction accuracy. 

## 4. Conclusions

A DNN model was developed for the prediction of *T*_g_ of PHA-based homo- and copolymers. The information of monomers is encoded by its digital fingerprints and subsequently inputs to the DNN model together with other factors affecting the *T*_g_. The hyperparameters of the DNN model are optimized with Bayesian optimization schema. The results indicate that the *T*_g_ values predicted by the DNN model are in good agreement with experimental results. The symmetry of the DNN model is achieved by incorporating the symmetry data set into the training data set, which further improves the model prediction performance. The interpretability analyses based on the importance factors revealed that the types of the two monomers are most important in determining the *T*_g_ values of the copolymers, while the size and dispersity of the polymers did not significantly affect the *T*_g_ values. Furthermore, different strategies to cope with missing data were evaluated, including removal of the input node, as well as estimation of the missing data with the mean, minimum, or maximum values of the data set. The results indicate that the DNN model is robust in achieving high accuracy even with missing data. Understanding the characteristics of the data distribution is important to making sound estimations of the missing data to ensure high performance of the DNN model. Compared with a commonly used ML model incorporating QSPR, the DNN model does not require an explicit descriptor selection step but shows a comparable performance. It is also noted that while the *T*_g_ is the targeted property in this study, the DNN model can be readily adapted to predict other properties of PHA homopolymers and copolymers.

## Figures and Tables

**Table 1:** The *R*^2^, MAE, and RMSE of the cross-validated subgroups and their averaged scores.

Subgroup	Subgroup 1	Subgroup 2	Subgroup 3	Subgroup 4	Subgroup 5	Average
*R* ^2^	0.799	0.883	0.857	0.824	0.914	0.869
MAE	3.792	3.970	4.976	3.745	3.541	4.010
RMSE	5.147	5.356	6.522	4.775	4.643	5.339

**Table 2:** Comparison of the results by the support vector machine (SVM), least absolute shrinkage and selection operator (LASSO) model, and DNN model.

Model	Subgroup	Subgroup 1	Subgroup 2	Subgroup 3	Subgroup 4	Subgroup 5	Average
SVM	*R* ^2^	0.684	0.828	0.766	0.791	0.778	0.783
	MAE	3.981	4.075	6.285	3.937	4.436	4.548
	RMSE	6.403	6.488	8.361	5.206	7.449	6.871
LASSO	*R* ^2^	0.607	0.802	0.768	0.648	0.610	0.714
	MAE	4.820	4.258	5.988	4.986	6.270	5.259
	RMSE	7.142	6.963	8.322	6.763	9.879	7.892
DNN	*R* ^2^	0.799	0.883	0.857	0.824	0.914	0.869
	MAE	3.792	3.970	4.976	3.745	3.541	4.010
	RMSE	5.147	5.356	6.522	4.775	4.643	5.339

**Table 3:** The *R*^2^, MAE, and RMSE with different ignored inputs.

Ignored Features	Complete Inputs	Fractional Composition of A	MW	PDI	MW and PDI
*R* ^2^	0.897	0.578	0.861	0.859	0.846
MAE	3.592	7.269	4.086	4.206	4.520
RMSE	4.812	9.586	5.511	5.543	5.800

**Table 4:** The descriptive statistics of the input data.

Statistics	Mean	SD*	Max	Min	Median
Fractional composition of A	67.1	31.5	100	0	74.5
MW	1159.8	1313.86	5240.4	47	505
PDI	2.6	1.1	6.9	1.1	2.5

SD* is the acronym standard deviation.

**Table 5:** The *R*^2^, MAE, and RMSE of the DNN model prediction when different input variables are estimated with their corresponding mean values.

Input Features	Complete Inputs	Fractional Composition of A	MW	PDI	MW and PDI
*R* ^2^	0.897	0.423	0.866	0.865	0.864
MAE	3.592	8.819	4.055	4.086	4.083
RMSE	4.812	11.215	5.410	5.416	5.448

**Table 6:** The *R*^2^, MAE, and RMSE with DNN model prediction when different input variables are estimated with their minimum values.

Input Features	Complete Inputs	Fractional Composition of A	MW	PDI	MW and PDI
*R* ^2^	0.897	−1.488	0.857	0.835	0.807
MAE	3.592	20.146	4.302	4.590	5.204
RMSE	4.812	23.290	5.575	5.994	6.494

**Table 7:** The *R*^2^, MAE, and RMSE with DNN model prediction when different input variables are estimated with their maximum values.

Input Features	Complete Inputs	Fractional Composition of A	MW	PDI	MW and PDI
*R* ^2^	0.897	0.053	0.711	0.688	0.210
MAE	3.592	10.512	6.383	6.937	11.474
RMSE	4.812	14.370	7.942	8.247	13.123

## Supplementary Materials

Available Statement: The following are available online at https://www.mdpi.com/1996-1944/13/24/5701/s1, Table S1: the polymer training dataset employed for the *T*_g_ prediction model development.
