# Supplementary material for: A Deep Neural Network for Accurate and Robust Prediction of the Glass Transition Temperature of Polyhydroxyalkanoate Homo- and Copolymers
Source: Materials (Basel). 2020 Dec 14;13(24):5701. doi: 10.3390/ma13245701 (PMC7765086; doi:10.3390/ma13245701)
Supplement: Supplementary file 1 [file materials-13-05701-s001.pdf]

materials

Supplementary Materials

A Deep Neural Network for Accurate and Robust Prediction of the Glass Transition Temperature of Polyhydroxyalkanoate Homo- and Copolymers

Table S1. The polymer training dataset employed for the T_g prediction model development.

Monomer A	Monomer B	Monomer A Ratio	Copolymer Nature	Mol. Weight (in 10^3)	PDI	T_g (K)	References
3H5PhP		100.0	R	350.0	3.50	287.15	[1]
3H5PhP	3H7PhHp	23.0	R	156.0	2.33	261.95	[2]
3H5PhP		100.0	R	79.0	2.20	288.85	[3]
3H6PhH		100.0	R	475.0	2.20	271.85	[2]
3H6PhH	3H4PhB	89.4	R	113.0	2.70	278.45	[3]
3H6PhH	3H8PhO	27.0	R	165.0	2.01	258.35	[2]
3HB		100.0	R	1623.0	1.22	276.15	[4]
3HB		100.0	R	166.0	2.60	277.15	[5]
3HB		100.0	R	3240.0	1.70	281.15	[6]
3HB	2HB	91.0	R	100.0	3.10	272.05	[7]
3HB	2HP	97.5	R	309.0	2.71	273.15	[7]
3HB	2HP	96.3	R	303.0	2.68	273.15	[7]
3HB	2HP	96.4	R	423.0	2.73	273.15	[7]
3HB	2HP	97.9	R	278.0	2.36	273.15	[7]
3HB	3H4MeV	60.4	B	1050.0	2.50	269.15	[8]
3HB	3H4MeV	67.7	B	1250.0	2.70	269.15	[8]
3HB	3H4MeV	73.8	B	1340.0	3.10	271.15	[8]
3HB	3HHx	89.0		346.0	1.22	271.57	[9]
3HB	3HP	24.0		652.4	2.80	262.15	[10]
3HB	3HP	33.0		704.0	3.20	263.15	[11]
3HB	3HP	29.0		704.0	3.20	262.15	[11]
3HB	3HP	54.0		684.6	6.52	273.15	[10]
3HB	3HP	22.0		420.0	3.00	259.15	[11]
3HB	3HP	12.0		319.0	2.90	258.15	[11]
3HB	3HP	75.0		208.1	2.19	273.15	[10]
3HB	3HP	80.0		588.0	2.10	272.15	[11]
3HB	3HP	89.0		735.0	2.10	274.15	[11]
3HB	3HP	57.0		672.0	2.40	268.15	[11]
3HB	3HP	63.0		638.0	2.20	268.15	[11]
3HB	3HP	93.0		704.0	2.20	276.15	[11]
3HB	3HP	71.0		609.0	2.90	271.15	[11]
3HB	3HP	85.0		382.0	2.29	277.15	[10]
3HB	3HV	55.0		960.0	2.40	263.15	[12]
3HB	3HV	54.0		450.0	2.30	267.40	[13]
3HB	3HV	36.0		3526.4	3.20	260.15	[14]
3HB	3HV	27.0		3324.6	3.11	262.15	[14]
3HB	3HV	29.0		508.0	2.00	260.15	[12]
3HB	3HV	32.0		410.0	2.00	266.55	[13]
3HB	3HV	68.0	R	550.0	1.40	266.55	[15]
3HB	3HV	35.0		2774.4	2.78	269.15	[14]
3HB	3HV	44.0		3452.7	3.08	272.15	[14]
3HB	3HV	12.0		4252.8	3.20	259.15	[14]
3HB	3HV	12.0		3857.2	3.03	259.15	[14]
3HB	3HV	8.0		4997.2	2.48	258.15	[14]
3HB	3HV	37.0		3286.1	2.88	272.15	[14]
3HB	3HV	13.1		3580.0	2.45	263.25	[16]
3HB	3HV	8.0		5240.4	2.64	258.15	[14]
3HB	3HV	6.0		4853.3	2.31	258.15	[14]
3HB	3HV	24.0		2930.4	2.91	270.15	[14]
3HB	3HV	5.0		4849.7	2.42	258.15	[14]
3HB	3HV	65.0	R	290.0	5.60	263.45	[15]
3HB	3HV	70.0	R	250.0	2.90	269.25	[15]

3HB	3HV	0.5		1690.0	2.17	255.28	[16]
3HB	3HV	71.0	R	410.0	1.40	269.45	[15]
3HB	3HV	37.0	R	1700.0	2.20	267.15	[6]
3HB	3HV	76.0		452.2	1.90	267.15	[12]
3HB	3HV	69.9	R	1730.0	1.43	270.04	[17]
3HB	3HV	22.0	R	1830.0	1.90	271.15	[6]
3HB	3HV	90.9	R	1560.0	1.41	275.40	[17]
3HB	3HV	88.0		2542.0	2.48	276.15	[14]
3HB	3HV	84.0		2608.6	2.56	276.15	[14]
3HB	3HV	87.0	R	1800.0	2.61	262.15	[18]
3HB	3HV	97.5	R	1120.0	1.07	276.55	[19]
3HB	3HV	100.0		2965.0	2.84	276.15	[14]
3HB	3HV	100.0		3620.0	3.06	276.15	[14]
3HB	3HV	100.0		3581.8	3.12	276.15	[14]
3HB	3HV	100.0		3173.1	2.96	276.15	[14]
3HB	3HV	97.4	R	1732.0	1.20	275.35	[19]
3HB	3HV	79.4		3400.0	2.48	272.65	[16]
3HB	3HV	57.0		3909.8	2.98	275.15	[14]
3HB	3HV	62.0		3733.9	2.89	274.15	[14]
3HB	3HV	28.5		3070.0	2.44	263.55	[16]
3HB	3HV	55.6		2710.0	1.76	267.65	[16]
3HB	3HV	66.7	B	2000.0	1.63	264.25	[17]
3HB	3HV	53.2	B	1470.0	1.31	265.85	[17]
3HB	4HB	17.0	R	126.0	2.80	233.15	[5]
3HB	4HB	24.0	R	126.0	4.10	236.15	[5]
3HB	4HB	60.0	R	793.0	2.50	257.15	[20]
3HB	4HB	9.0	R	60.0	1.54	220.15	[21]
3HB	4HB	46.0	R	165.0	3.10	251.15	[5]
3HB	4HB	11.0	R	61.0	1.49	218.15	[21]
3HB	4HB	66.0	R	150.0	2.80	270.15	[5]
3HB	4HB	15.0	R	87.0	2.23	232.15	[21]
3HB	4HB	35.0	R	47.0	1.82	242.15	[22]
3HB	4HB	17.0	R	98.0	2.09	232.15	[21]
3HB	4HB	70.0	R	376.0	3.55	256.15	[22]
3HB	4HB	80.0	R	391.0	3.60	257.15	[21]
3HB	4HB	79.0	R	375.0	3.10	257.15	[22]
3HB	4HB	71.0	R	110.0	5.00	270.15	[5]
3HB	4HB	90.0	R	342.0	2.63	261.15	[22]
3HB	4HB	89.6		570.0	3.80	276.55	[23]
3HB	4HB	89.0		320.0	2.67	269.15	[24]
3HB	4HB	94.2	R	1800.0	3.27	255.15	[18]
3HB	4HB	87.0	R	153.0	2.80	272.15	[5]
3HB	4HB	95.7	R	1300.0	3.10	273.15	[18]
3HB	4HB	87.0	R	74.0	5.00	269.15	[5]
3HB	4HB	85.0		750.0	3.00	270.55	[23]
3HB	4HB	92.0	R	88.0	6.20	272.15	[5]
3HB	4HB	90.6		380.0	1.81	269.15	[24]
3HB	4HB	94.8	R	1700.0	3.15	253.15	[18]
3HB	4HB	95.3		580.0	2.52	273.15	[24]
3HB	4HB	97.0	R	58.0	6.90	274.15	[5]
3HB	4HB	100.0	R	2200.0	3.44	277.15	[18]
3HB	4HB	100.0		640.0	2.56	276.15	[24]
3HB	4HB	19.7	B	50.0	3.10	225.85	[25]
3HB	4HB	65.0	R	351.0	6.20	253.15	[21]
3HB	4HB	61.0	R	330.0	5.19	250.15	[22]
3HB	4HB	53.0	R	51.0	1.74	243.15	[22]
3HB		100.0		1635.0	1.93	270.07	[9]
3HB		100.0	R	153.0	3.30	281.05	[3]
3HB		100.0	R	505.0	1.87	275.65	[22]
3HB		100.0		226.0	1.13	275.65	[26]
3HB		100.0	R	2200.0	3.44	277.15	[18]
3HB		100.0		1459.2	1.90	277.15	[11]
3HD		100.0	R	361.0	1.45	235.94	[27]
3HH		100.0	R	272.0	1.93	244.96	[27]
3HHp		100.0		455.0	1.81	241.02	[9]
3HHx		100.0		272.0	1.32	244.96	[9]
3HO	3HB	98.0		180.0	1.22	234.77	[9]
3HP	4HB	18.2		302.0	1.28	243.67	[28]
3HP	4HB	88.1		284.0	1.29	248.73	[28]

3HP	4HB	74.5		259.0	1.32	241.87	[28]
3HP	4HB	62.1		280.0	1.30	237.01	[28]
3HP	4HB	33.0		278.0	1.31	231.28	[28]
3HP	4HB	63.0	B	209.0	2.62	239.77	[29]
3HP	4HB	71.0	B	217.0	2.52	239.75	[29]
3HP	4HB	75.0		172.0	2.83	241.56	[29]
3HP		100.0		200.2	1.40	254.15	[11]
3HP		100.0		163.0	1.48	255.30	[28]
3HV		100.0		230.0	3.50	257.35	[26]
3HV		100.0		1056.0	1.30	258.06	[9]
4HB		100.0		854.0	1.75	227.48	[9]
4HB		100.0		389.0	1.17	226.15	[28]

Reference

1. Fritzsche, K.; Lenz, R.W.; Fuller, R.C. An unusual bacterial polyester with a phenyl pendant group. *Macromol. Chem. Phys.* **1990,** *191*, 1957–1965.
2. Abraham, G.A.; Gallardo, A.; Roman, J.S.; Olivera, E.R.; Jodra, R.; Garc´ıa, B.; Min˜ambres, B.; Garc´ıa, J.L.; Luengo, J.M. Microbial synthesis of poly(beta-hydroxyalkanoates) bearing phenyl groups from pseudomonas putida: chemical structure and characterization. *Biomacromolecules,* **2001**, *2*, 562, ISSN 1525–7797.
3. Mizuno, S.; Katsumata, S.; Hiroe, A.; Tsuge, T. Biosynthesis and thermal characterization of polyhydroxyalkanoates bearing phenyl and phenylalkyl side groups. *Polym. Degrad. Stab.*, **2014**, *109*, 379, ISSN 0141–3910.
4. Luo R.; Chen J.; Zhang L.; Chen G. Polyhydroxyalkanoate copolyesters produced by Ralstonia eutropha PHB−4 harboring a low-substrate-specificity PHA synthase PhaC2Ps from Pseudomonas stutzeri 1317. *Biochem. Eng. J.*, **2006**, *32*, 218, ISSN 1369–703X.
5. Kang, C.-K.; Kusaka, S.; Doi, Y. Structure and properties of poly(3-hydroxybutyrate-co-4-hydroxybutyrate) produced by *Alcaligenes latus*. *Biotechnol Lett*, **1995**, *17*, 583–588, ISSN 1573–6776.
6. Myung, J.; Flanagan, J.C.A.; Waymouth, R.M.; Criddle, C.S. Methane or methanol-oxidation dependent synthesis of poly(3-hydroxybutyrate-co-3-hydroxyvalerate) by obligate type II methanotrophs. *Process. Biochem.*, **2016**, *51*, 561, ISSN 1359–5113.
7. Han, X.; Satoh, Y.; Satoh, T.; Matsumoto, K.; Kakuchi, T.; Taguchi, S.; Dairi, T.; Munekata, M.; Tajima, K. Chemo-enzymatic synthesis of polyhydroxyalkanoate (PHA) incorporating 2-hydroxybutyrate by wild-type class I PHA synthase from *Ralstonia eutropha. Appl Microbiol Biotechnol,* **2011,** *92*, 509–517, ISSN 1432–0614.
8. Tanadchangsaeng, N.; Kitagawa, A.; Yamamoto, T.; Abe, H.; Tsuge, T. Identification, Biosynthesis, and Characterization of Polyhydroxyalkanoate Copolymer Consisting of 3-Hydroxybutyrate and 3-Hydroxy-4-methylvalerate. Biomacromolecules, **2009**, *10*, 2866–2874, ISSN 1525–7797.
9. Wang, H.-h.; Zhou, X.-r.; Liu, Q. Chen, G.-Q. Biosynthesis of polyhydroxyalkanoate homopolymers by Pseudomonas putid. *Appl Microbiol Biotechnol*, **2011**, *89*, 1497, ISSN 1432–0614.
10. Na, Y.-H.; He, Y.; Asakawa, N.; Yoshie, N.; Inoue, Y. Miscibility and Phase Structure of Blends of Poly(ethylene oxide) with Poly(3-hydroxybutyrate), Poly(3-hydroxypropionate), and Their Copolymers. *Macromol.*, **2002**, *35*, 727–735, ISSN 0024–9297.
11. Shimamura, E.; Scandola, M.; Doi, Y. Microbial Synthesis and Characterization of Poly(3-Hydroxybutyrate-Co-3-Hydroxypropionate). *Macromol.*, **1994**, *27*, 4429–4435, ISSN 0024–9297.
12. Chanprateep, S.; Kulpreecha, S. Production and characterization of biodegradable terpolymer poly(3-hydroxybutyrate-co-3-hydroxyvalerate-co-4-hydroxybutyrate) by Alcaligenes sp. A-04. *J. Biosci. Bioeng.*, **2006**, *101*, 51–56, ISSN 1389–1723.
13. Laycock, B.; Arcos-Hernandez, M.V.; Langford, A.; Buchanan, J.; Halley, P.J.; Werker, A.; Lant, P.A.; Pratt, S. Thermal properties and crystallization behavior of fractionated blocky and random polyhydroxyalkanoate copolymers from mixed microbial cultures. *J. Appl. Polym. Sci.*, **2014**, *131*, ISSN 1097–4628.
14. Ashby, R.D.; Solaiman, D.K.Y.; Nun˜ez, A.; Strahan, G.D.; Johnston, D.B. Burkholderia sacchari DSM 17165: A source of compositionally-tunable block-copolymeric short-chain poly (hydroxyalkanoates) from xylose and levulinic acid. *Bioresour. Technol.*, **2018**, *253*, 333, ISSN 0960–8524.

15. Dai, Y.; Yuan, Z.; Jack, K.; Keller, J. Production of targeted poly (3-hydroxyalkanoates) copolymers by glycogen accumulating organisms using acetate as sole carbon source. *J. Biotechnol.*, **2007**, *129*, 489–497, ISSN 0168–1656.
16. Ferre-Guell, A.; Winterburn, J. Biosynthesis and characterization of polyhydroxyalkanoates with controlled composition and microstructure. *Biomacromolecules*, **2018**, *19*, 996, ISSN 1525–7797.
17. Han, J.; Wu, L.-P.; Hou, J.; Zhao, D.; Xiang, H. Biosynthesis, Characterization, and Hemostasis Potential of Tailor-Made Poly(3-hydroxybutyrate-co-3-hydroxyvalerate) Produced by *Haloferax mediterranei*. *Biomacromolecules*, **2015**, *16*, 578–588, ISSN 1525–7797.
18. Phong, T.H.; Van Thuoc, D.; Sudesh, K. Biosynthesis of poly (3-hydroxybutyrate) and its copolymers by Yangia sp. ND199 from different carbon sources. *Int. J. Biol. Macromol.*, **2016**, *84*, 361–366, ISSN 0141–8130.
19. Povolo, S.; Romanelli, M.G.; Basaglia, M.; Ilieva, V.I.; Corti, A.; Morelli, A.; Chiellini, E.; Casella, S. Polyhydroxyalkanoate biosynthesis by Hydrogenophaga pseudoflava DSM1034 from structurally unrelated carbon sources. *New Biotechnol.*, **2013**, *30*, 629–634, ISSN 1871–6784.
20. Huong, K.-H.; Azuraini, M.J.; Aziz, N.A.; Amirul, A.-A.A. Pilot scale production of poly (3-hydroxybutyrate-co-4-hydroxybutyrate) biopolymers with high molecular weight and elastomeric properties. *J. Biosci. Bioeng.*, **2017**, *124*, 76–83, ISSN 1389–1723.
21. Syafiq, I.M.; Huong, K.-H.; Shantini, K.; Vigneswari, S.; Aziz, N.A.; Amirul, A.-A.A.; Bhubalan, I. Synthesis of high 4-hydroxybutyrate copolymer by Cupriavidus sp. transformants using one-stage cultivation and mixed precursor substrates strategy. *Enzym. Microb. Technol.*, **2017**, *98*, 1–8, ISSN 0141–0229.
22. Iqbal, N.M.; Amirul, A.A. Synthesis of P(3HB-co-4HB) copolymer with target-specific 4HB molar fractions using combinations of carbon substrates. *J. Chem. Technol. Biotechnol.*, **2014**, *89*, 407, ISSN 1097–4660.
23. Zhila, N.; Shishatskaya, E. Properties of PHA bi-, ter-, and quarter-polymers containing 4-hydroxybutyrate monomer units. *Int. J. Biol. Macromol.*, **2018**, *111*, 1019–1026, ISSN 0141–8130.
24. Zhang, J.; Kasuya, K.; Hikima, T.; Takata, M.; Takemura, A.; Iwata, T. Mechanical properties, structure analysis and enzymatic degradation of uniaxially cold-drawn films of poly [(R)-3-hydroxybutyrate-co-4-hydroxybutyrate]. *Polym. Degrad. Stab.*, **2011**, *96*, 2130–2138, ISSN 0141–3910.
25. Hu, D.; Chung, A.-L.; Wu, L.-P.; Zhang, X.; Wu, Q.; Chen, J.-C.; Chen, G.-Q. Biosynthesis and Characterization of Polyhydroxyalkanoate Block Copolymer P3HB-b-P4HB. *Biomacromolecules*, **2011**, *12*, 3166–3173, ISSN 1525–7797.
26. Shen, X.-W.; Yang, Y.; Jian, J.; Wu, Q.; Chen, G.-Q. Production and characterization of homopolymer poly (3-hydroxyvalerate)(PHV) accumulated by wild type and recombinant Aeromonas hydrophila strain 4AK4. *Bioresour. Technol.*, **2009**, *100*, 4296–4299, ISSN 0960–8524.
27. Tripathi, I.; Wu, L.-P.; Dechuan, M.; Chen, J.; Wu, Q.; Chen, G.-Q. Pseudomonas putida KT2442 as a platform for the biosynthesis of polyhydroxyalkanoates with adjustable monomer contents and compositions. *Bioresour. Technol.*, **2013**, *142*, 225–231, ISSN 0960–8524.
28. Meng, D.-C.; Shi, Z.-Y.; Wu, L.-P.; Zhou, Q.; Wu, Q.; Chen, J.-C.; Chen, G.-Q. Production and characterization of poly(3-hydroxypropionate-co-4-hydroxybutyrate) with fully controllable structures by recombinant Escherichia coli containing an engineered pathway. *Metab. Eng.*, **2012**, *14*, 317, ISSN 1096–7176.
29. Tripathi, M.; Wu, L.-P.; Meng, D.; Chen, J. Chen, G.-Q. Biosynthesis and Characterization of Diblock Copolymer of P(3-Hydroxypropionate)-block-P(4-hydroxybutyrate) from Recombinant *Escherichia coli*. *Biomacromolecules*, **2013**, *14*, 862–870, ISSN 1525–7797.

Publisher's Note: MDPI stays neutral with regard to jurisdictional claims in published maps and institutional affiliations.

© 2020 by the authors. Submitted for possible open access publication under the terms and conditions of the Creative Commons Attribution (CC BY) license (http://creativecommons.org/licenses/by/4.0/).
